# Transcriptional plasticity of fibroblasts in heart disease

**DOI:** 10.1042/BST20210864

**Published:** 2022-10-25

**Authors:** Rudi Micheletti, Michael Alexanian

**Affiliations:** 1 Howard Hughes Medical Institute, Department and School of Medicine, University of California, San Diego, La Jolla, CA, U.S.A.; 2 Gladstone Institutes, San Francisco, CA, U.S.A.; 3 Department of Pediatrics, University of California, San Francisco, CA, U.S.A.

**Keywords:** epigenomics, fibroblasts, heart disease

## Abstract

Cardiac fibroblasts play an essential role in maintaining the structural framework of the heart. Upon stress, fibroblasts undergo a cell state transition to activated fibroblasts (also referred to as myofibroblasts), a highly synthetic cell type that proliferates, migrates, and secrets both extracellular matrix as well as signaling factors that can modulate cellular crosstalk [*J. Clin. Invest.* 132, e148554]. Activated fibroblasts are critical regulators of cardiac wound healing after injury, but their excessive and persistent activation promote tissue fibrosis, a hallmark feature of the pathological remodeling of the heart. While much of the previous work in cardiac fibroblast biology has focused on the role of canonical signaling pathways or components of the extracellular matrix, recent efforts have been focused on deciphering the gene regulatory principles governing fibroblast activation. A better understanding of the molecular mechanisms that trigger and sustain the fibrotic process in heart disease has the potential to accelerate the development of therapies that specifically target the cardiac activated fibroblasts, which are at the moment unavailable. This concise review focuses on the mechanisms underlying the chromatin and transcriptional regulation of cardiac fibroblast activation. We discuss recent work from our group and others in this space, highlighting the application of single-cell genomics in the characterization of fibroblast function and diversity, and provide an overview on the prospects of targeting cardiac fibroblasts in heart disease and the associated challenges.

## Introduction

Fibrosis is a stereotypical wound-healing response that is mounted after tissue injury or stress and is often associated with organ dysfunction in a variety of human diseases. In the context of heart disease, the leading cause of mortality in the developed world, a significant body of clinical data has demonstrated that cardiac fibrosis is strongly associated with adverse outcomes in several conditions, including heart failure with reduced ejection function (HFrEF), heart failure with preserved ejection function (HFpEF), and genetically driven cardiomyopathies [1]. While the acute fibrotic responses can stabilize a focal area of myocardial damage, clinical and experimental studies support the notion that chronic and uncontrolled activation of the fibrotic process can be deleterious to long-term cardiac function and patient survival. Resident fibroblasts, which make up ∼20% of the non-myocytes in the heart [2], are the central mediators of the fibrotic response in this organ. In response to cardiac injury and stress, fibroblasts are activated and acquire a profibrotic phenotype. While activated fibroblasts have long been suspected to participate in heart disease pathogenesis, defining a causal role for this cell type has been historically challenging due to the lack of robust tools that would allow for *in vivo* interrogations of this cell population. These limitations have been circumvented following the generation of powerful new mouse models [3], which have demonstrated that ablation of activated fibroblasts [4] or the deletion of proximal regulators of cardiac fibroblast activation [5] (e.g. transforming growth factor-β (TGF-β) receptors) can block the fibrotic response and attenuate heart disease progression *in vivo*, providing cogent evidence that the activated fibroblast is a major driver of disease pathogenesis.

Understanding the mechanistic basis that governs the switch from quiescent to activated fibroblast states remains a major unresolved question in the field of cardiac fibroblast biology with important therapeutic implications.

## TGF-β is a master signaling pathway for fibroblast activation

Several signaling pathways have been implicated in the regulation of cardiac fibroblast activation and fibrosis. Among these, the canonical and noncanonical TGF-β signaling, the adrenergic receptor and the renin-angiotensin-aldosterone systems (reviewed extensively elsewhere [1]). Within this context, the role of TGF-β signaling in governing cardiac fibroblast activation has been extensively studied both *in vitro* and *in vivo*. In response to cardiac stress or injury, TGF-β is activated to trigger fibroblast proliferation, migration, contractility and ECM deposition [6]. Several studies have explored the role of the TGF-β-signaling transcriptional mediators, SMADs, in governing the gene programs that control fibroblast activation. Initial *in vivo* studies with SMAD3 global deletion demonstrated a reduction in myocardial fibrosis across several models of murine heart failure [7, 8]. More recently, activated fibroblast-specific deletion of *Tgfbr1/2* or *Smad3*, but not *Smad2,* was shown to reduce cardiac fibrosis in a model of pressure overload-induced heart failure (transverse aortic constriction, TAC), confirming that TGF-β signaling is a nodal regulator of profibrotic function in the heart [5]. Of note, parallel studies using the same rodent heart failure model showed that activated fibroblast-specific *Smad3* deletion is also associated with detrimental effects in cardiac function [9], highlighting the complexity of TGF-β signaling in mediating the response to cardiac stress. Apart from SMAD-mediated transcription, TGF-β can also initiate non-canonical signaling that leads to MAPK cascade activation and ultimately to p38, JNK1/2, and ERK1/2 signaling [10]. Within this context, fibroblast-specific deletion or overexpression of p38α impaired or increased cardiac fibrotic responses, respectively [11]. Further excitement about the possibility of targeting MAPK/p38 signaling as an anti-fibrotic approach is provided by recent data using the drug salinomycin to treat pre-established cardiac fibrosis [12]. However, given the broad expression of these kinases coupled with their divergence in tissue function and regulation, the ability to deliver such inhibitors specifically to fibroblasts will be key to avoiding on-target toxicity in off-target cell types.

## Stress signals converge on the fibroblast epigenomic machinery

The stress-induced pathways that trigger fibroblast activation ultimately converge on transcription factors (TFs) and the chromatin regulatory apparatus in the nucleus, which transduce these broad upstream signals into changes in gene expression and cell identity. Fibroblast activation, like any other cell state transition, is initiated by the binding of TFs and chromatin regulators at the level of the DNA, which triggers the activation and repression of a defined set of enhancers. Among the epigenetic modifications, acetylation of lysine residues (Kac) on histone tails is a major feature of chromatin activation. Importantly, previous studies have demonstrated a critical role of histone acetyltransferases and histone deacetylases in controlling cardiac profibrotic function (extensively reviewed elsewhere [1]). Context-specific recognition of Kac at regions of actively transcribed euchromatin is mediated by epigenetic reader proteins possessing a Kac-recognition module, or bromodomain. Of these bromodomain-containing reader proteins, the BET (Bromodomain and Extra-Terminal) family, which comprise the ubiquitously expressed BRD2, BRD3, and BRD4 along with the testis-specific BRDT, has become the subject of intense study given their role as transcriptional coactivators in a variety of diseases. The ability to probe the function of BET proteins has been accelerated by the use of the small molecule compound JQ1 [13], a potent and specific BET inhibitor that competitively and reversibly displaces BETs from their endogenous targets, thereby disrupting BET-dependent transcription. Numerous studies in the last decade established proof of principle that BET reader proteins are essential coactivators of stress-dependent gene expression programs in heart failure pathogenesis, and that their inhibition robustly ameliorates heart failure across a variety of mouse models [14–16]. The precise identity of cell-types that mediate these therapeutic effects *in vivo* remains an unanswered question with important therapeutic implications.

## BET proteins regulate a reversible transcriptional switch that governs fibroblast activation

The use of small molecule compounds that reversibly inhibit stress-dependent transcription *in vivo*, coupled with single-cell genomics, offers the unique opportunity to interrogate which cell types, gene programs and molecular mechanisms are dynamically regulated in disease pathogenesis. To explore these approaches in the context of the TAC model of pressure-overload heart failure, we have recently combined the use of single-cell RNA- and ATAC-sequencing (scRNA-seq, scATAC-seq) in the setting of intermittent BET bromodomain inhibitor exposure [17]. In this context, one month of JQ1 treatment in pre-established murine heart failure significantly improves left ventricular systolic function, with JQ1 withdrawal leading to a rapid regression, suggesting that BET bromodomain inhibition is characterized by therapeutic reversibility [17]. Our scRNA-seq analysis highlighted that the transcriptomes of resident cardiac fibroblasts robustly toggled between quiescent and activated states in a manner that directly correlated with BET inhibitor exposure and cardiac function. As BET proteins are known chromatin regulators, we hypothesized that the observed transcriptional reversibility from JQ1 exposure would correlate with changes in chromatin accessibility and enhancer activation in cardiac fibroblasts during heart failure pathogenesis. With this in mind, we developed a novel approach to correlate single-cell chromatin accessibility with organ function that revealed a set of genomic regions anticorrelated with heart failure severity. These distal regions demonstrated increased chromatin accessibility following TAC, decreased accessibility with JQ1 treatment, and an attendant increase in accessibility when the BET inhibitor treatment was stopped.

## MEOX1 as a potential switch for controlling stress-dependent fibroblast activation

One of the enhancer elements whose chromatin accessibility was most anticorrelated with left ventricular systolic function was a large enhancer proximal to *Meox1*, a poorly studied TF known to control sclerotome polarization during development [18]. The expression of *Meox1* was also dynamically regulated in the setting of stress and exposure to JQ1: strongly increased in fibroblasts upon TAC, abolished by JQ1 treatment, and robustly induced again upon JQ1 withdrawal [17]. The large putative enhancer proximal to *Meox1* is characterized by multiple distal accessible regions, with several of these being sensitive to TAC and BET inhibition. As enhancers are pervasively transcribed and nascent transcription is a robust indicator of enhancer activity [19], we performed precision nuclear run-on sequencing (PROseq) on cultured fibroblasts *in vitro* to map genome-wide RNA polymerase II nascent transcription and identify putative active enhancers. This highlighted that within the large regulatory element proximal to *Meox1,* one specific region (which we named *Peak9/10* enhancer) was associated with a strong increase in nascent transcription following TGF-β-induced fibroblast activation *in vitro*. We used CRISPR interference to repress a series of distal elements within the *Meox1* regulatory region and found that the *Peak9/10* enhancer was specifically required for *Meox1* transactivation upon TGF-β stimulation, while other accessible regions identified *in vivo* were not [17]. This highlighted the power of coupling measurements of chromatin accessibility with sensitive assays of enhancer activity to define which accessible DNA elements may serve as functionally relevant regulatory elements.

To understand the role of *Meox1* as a putative regulator of stress-dependent fibroblast activation, we performed a series of *in vitro* assays that demonstrated how *Meox1* depletion in TGF-β treated cells decreased the formation of smooth muscle actin (SMA)-positive stress fibers, collagen-gel contraction and EdU incorporation, suggesting that MEOX1 was required for contractile and proliferative phenotypic transitions *in vitro*, two functional hallmarks of stress-activated fibroblasts. Finally, we combined MEOX1 ChIPseq with PROseq in the context of *Meox1* depletion to demonstrate that upon fibroblast activation, MEOX1 binds and directly regulates the transcription of a specific set of stress-responsive distal and gene elements. These findings suggest MEOX1 functions as a transcriptional mediator of the cell state transition between quiescent and activated fibroblast *in vitro*. Future studies are needed to explore the role of MEOX1 *in vivo* to understand if this TF is a critical regulator of fibroblast activation and fibrosis in heart failure pathogenesis. Of note, recent single-cell studies exploring the transcriptional landscape of the human heart in healthy and diseased tissues highlighted how *MEOX1* is expressed in human cardiac fibroblasts and up-regulated during disease progression [20, 21]. It is certainly tempting to speculate that MEOX1 could represent an ideal therapeutic target to inhibit fibrosis *in vivo*. Though, TFs have historically been considered undruggable due to their structure and complex functions. Within this context, there has been particular excitement around the possibility of using proteolysis targeting chimeras (PROTACs) [22] to target TFs for degradation. Future innovations in drug development might finally allow efficient targeting of TFs involved in fibroblast activation and fibrosis.

## Fibroblast transcription in disease at single-cell resolution

Single-cell genomics offers the unprecedented opportunity to unbiasedly capture the transcriptional landscape of complex tissues such as the heart. Several studies have recently used single-cell approaches to investigate fibroblast transcriptional heterogeneity in cardiac pathologies. Single-nucleus RNA-sequencing (snRNA-seq) on cardiac biopsies from controls and patients with dilated cardiomyopathy (DCM) or hypertrophic cardiomyopathy (HCM) has highlighted how, amongst all cellular compartments, the fibroblasts were the most transcriptionally dysregulated in patients with cardiomyopathy [23]. Indeed, patients with DCM and HCM possessed a unique population of activated fibroblasts that was almost entirely absent from healthy samples, characterized by the expression of a specific gene set including known activated fibroblast markers such as *POSTN* and *FAP* as well as previously unreported genes such as *NALF1* and *TSHZ2*. An elegant *in vitro* CRISPR interference assay coupled with immunofluorescence to measure changes in SMA content revealed how the depletion of a subset of these genes decreased profibrotic response. Another recent study has utilized snRNA-seq on cardiac specimens obtained from human donors affected by different types of congenital heart disease (CHD), including hypoplastic left heart syndrome (HLHS) and Tetralogy of Fallot (TOF) [21]. Again, the authors identified a subset of activated cardiac fibroblasts derived exclusively from CHD patients characterized by the expression of genes including *POSTN*, *FAP* and *MEOX1*.

Several studies have also sought to characterize fibroblast transcriptional heterogeneity and dynamics in murine models of heart failure. ScRNA-seq in non-myocyte *Pdgfra* positive cells at days 3 and 7 post myocardial infarction (MI) identified novel fibroblast cell states, including a population characterized by the expression of canonical WNT pathway inhibitors along with several stress-activated fibroblasts subtypes with pro- or anti-fibrotic signatures [24]. Similarly, Forte et al. employed a genetic reporter driven by *Wt1* to sort epicardial-derived cells and performed scRNA-seq at several time-points (days 1, 3, 5, 7, 14 and 28) post-MI in two inbred strains, C57BL/6J and 129S1/SvImJ [25]. This dataset highlighted a dynamic transcriptional transition of fibroblasts upon injury: an acute response (days 1 and 3) characterized by a signature of reduced oxidative stress and immunomodulation; a proliferative phase (days 3, 5 and 7); and a later stage (days 14 and 28) marked by expression of downstream targets of TGF-β and ECM components involved in pathological remodeling. The comparison of the transcriptional response at day 3 post-MI in these two mouse strains, which are known to display a marked difference in the frequency of cardiac rupture, suggested that the increased susceptibility to rupture in the 129S1/SvImJ strain may be due to a prolonged fibroblast acute response, earlier fibroblast activation, and increased collagen deposition. This study represents an elegant example of how a divergent transcriptional response to injury can predict a phenotypic response to injury.

Another study dissected the fibroblast transcriptional changes in response to angiotensin II (AngII) infusion, another established murine model of cardiac hypertrophy [26]. Interestingly, the authors highlight two previously undescribed profibrotic populations, marked by the expression of *Cilp* and *Thbs4*, that do not correspond to the classical SMA–expressing activated fibroblasts. Notably, another group has recently used scRNA-seq in the TAC model of pressure overload and identified a cardiac fibroblast subpopulation which expands upon injury that acquires *Thbs4* expression [27]. While these works have identified novel stress-activated fibroblast subsets, the physiological relevance of these cell populations in disease pathogenesis remains to be tested. Finally, most of the single-cell studies have captured cardiac fibroblast states in the context of murine models of heart failure associated with systolic dysfunction. Future work using models of diastolic dysfunction to induce HFpEF could elucidate specific transcriptional and epigenomic changes of fibroblasts associated to these conditions.

## Feasibility and challenges in targeting cardiac fibroblasts in heart disease

**Figure 1.**
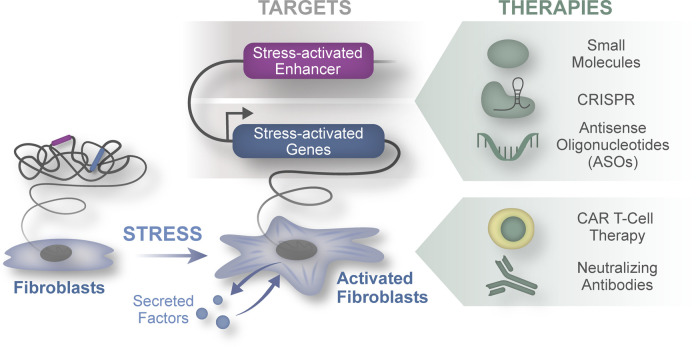
Promising therapeutic modalities to target fibroblasts in heart disease. Schematic illustration of the modalities that may be amenable to targeting fibroblasts in heart disease.

Fibroblast biology is complex and fascinating as these cells must maintain tissue architecture and participate in organ homeostasis across diverse organ systems. Fibroblasts are critical regulators of repair and wound healing, but their chronic activation results in tissue fibrosis, a finding that often correlates with organ dysfunction. Importantly, fibrosis is a shared feature across a variety of cardiac pathologies ranging from ischemic to inflammatory heart disease and represents a final common pathway in the development of heart failure regardless of the underlying etiology. Furthermore, the presence of patchy scar within the myocardium further predisposes patients to additional comorbid conditions including ventricular arrhythmias and sudden cardiac death. Given the central role of fibrosis across these diverse conditions, it is appealing to speculate that the development of anti-fibrotic therapies has the potential to benefit a broad swath of patients suffering from cardiac disease. While the notion of decreasing or depleting activated fibroblasts may represent a rational therapeutic strategy for treating a variety of heart disease (Figure 1), many challenges are associated with the feasibility of these approaches.

### The issue of specificity

Finding tissue and organ specific fibroblast features has historically been a significant challenge. As these cells reside across tissues with varied and distinct functions, it is likely that their ability to accomplish diverse tasks is, in part, due to defined tissue-specific gene regulatory networks. Consistent with the fact that organ fibroblasts are not generated from a common progenitor pool but instead arise independently during organogenesis, a recent single-cell study showed that organ fibroblasts possess defined molecular blueprints characterized by the expression of developmental TFs, such as *Tbx20* in the heart [28]. Other studies have focused on capturing fibroblast gene expression across tissues and found that, despite their heterogeneity, this lineage can be distinguished into universal and specialized subsets at steady-state with an activated subset arising in the context of disease [29]. These findings support the notion that mechanisms controlling fibroblast activation in one disease setting may potentially be translatable to other chronic diseases that prominently feature similar processes. While these studies have exponentially increased our understanding of the fibroblast transcriptional landscape, additional work is needed to further characterize fibroblasts subclusters, particularly in disease pathogenesis, with the hope of identifying functionally relevant cell states with a defined transcriptional profile. This is of particular importance as there remains a dearth of tissue- and stress-specific fibroblasts markers. As quiescent fibroblasts are critical in the maintenance of tissue architecture and participate actively in organ homeostasis, therapies aimed at this cell population must judiciously avoid interference with these activities. Indeed, identifying potential therapeutic targets that are both organ- and stress-specific will be one the greatest challenges in fibroblast biology over the next decade.

### Targeting fibroblast enhancers?

Given the challenges associated with identifying tissue-specific fibroblasts markers, it is possible to speculate that the non-coding genome could provide more tissue-restricted targets that can be explored for therapeutic manipulation. Indeed, *cis* regulatory elements possess increased tissue-specific activity as compared with protein coding genes [30] and are potent regulators of gene expression. Our recent work identified a class of fibroblast-enriched enhancers whose activation is anticorrelated with cardiac function, with a particular focus on one specific DNA element that we demonstrated can control the expression of the TF MEOX1 [17]. Apart from this specific region, we discovered hundreds of other dynamically accessible distal elements which we think represent exciting candidates for further study.

An enormous challenge in the translational potential for enhancer research lies in how to therapeutically target these regions. We and others have used BET inhibitors to target fibroblast enhancers and inhibit gene expression [17, 31]. Although these compounds are powerful tools to interdict stress-activated enhancer signaling *in vivo*, their use is associated with on-target toxicity in off-target tissues due to the broad expression of BET proteins. Further mechanistic refinement of the modalities by which downstream BET targets contribute to fibroblast activation will offer opportunities to develop therapeutic approaches that are tailored to targeted gene regulation specifically in the fibroblast cell compartment. In parallel, studies exploring the use of more specific BET inhibitors (e.g. those specifically targeting the first or second bromodomain [32, 33]) in murine models of heart disease have the potential to recapitulate the effects of compounds like JQ1 with regard to fibroblast gene expression in the setting of small molecule compounds with more favorable toxicity profiles. CRISPR-based therapies certainly represent another promising area for exploration in targeting enhancers implicated in fibroblast activation. The versatility of CRISPR-based therapies is intriguing, as they offer the potential for precise enhancer targeting that may be used to edit, inhibit, or activate a specific sequence. Another modality that may be amenable to targeting fibroblast-specific enhancers involves exploiting their transcription. Indeed, most if not all active enhancers are bi-directionally transcribed into short and non-coding RNAs [34, 35]. A small subset of enhancers is instead associated with the transcription of unidirectional, multi-exonic, alternatively spliced, and poly-adenylated transcripts (enhancer-associated lncRNAs or elncRNAs) [36, 37]. We have previously identified and described *Wisper* (Wisp2 super-enhancer-associated RNA), the first functionally relevant cardiac fibroblast-specific long non-coding RNA [38]. *Wisper* was up-regulated following MI in mice, has a human ortholog and its depletion *in vivo* using an antisense oligonucleotide (ASO; which recruits RNAse H to cleave and degrade the target RNA) was associated with marked reduction in cardiac fibrosis and a significant improvement in left ventricular systolic function. Importantly, *Wisper* expression was not detected in lung or kidney fibroblasts, supporting the notion that *Wisper* may have specificity for mediating cardiac fibrosis. Indeed, the ASO field is an emerging area of drug development that offers a promising alternative to targeting enhancers implicated in fibroblast activation. Targeting transcripts such *Wisper* that possess tissue- and cell-specificity could represent a valid approach to therapeutically target stress-activated cardiac fibroblasts with systemic delivery of nucleic acid-based therapies to extrahepatic tissues remaining one of the greatest barriers. The exquisite specificity of enhancer activity, when combined with their role as highly specialized nodal regulators of gene expression, make these genomic elements an incredibly attractive therapeutic target for heart disease.

### Ablating cardiac fibroblasts?

The concept that aberrant cell states can be targeted for ablation is well-established in cancer biology. Chimeric antigen receptor (CAR) T-cell therapy is a new form of immunotherapy that utilizes specially altered T-cells to target and eliminate specific cancer cells. In recent years, there has been growing interest in leveraging this therapeutic concept to non-oncology settings, including heart disease. Recent work from Jonathan Epstein's laboratory has been pivotal in establishing the proof-of-concept that CAR T-cell therapy can be used to target cardiac fibroblasts to reduce fibrosis and improve heart function in murine models of heart failure [39]. These authors have targeted and depleted fibroblast expressing FAP (fibroblast activation protein), a marker known to be up-regulated with cardiac stress [40]. More recently, the success of the COVID-19 mRNA lipid nanoparticle (LNP) vaccines has opened up immense possibilities for using LNP-based mRNA delivery for immunotherapy. Within this context, a very recent study has used mRNA encoding for FAP-CAR packaged into LNP coated with CD5-targeting antibodies that specifically target T cells to diminish cardiac fibrosis [41]. As fibroblast activation is part of a normal wound-healing process in many tissues, the transient nature of this approach, where no T cell remain persistently CAR positive, is particularly appealing as it avoids long-term targeting of cells that are critical for homeostatic processes. However, before these approaches can be translated to humans, much work remains to be done in assessing whether FAP marks the optimal cardiac fibroblast population to target for ablation. In this regard, the aforementioned advances in single-cell genomics could help identify alternative antigens expressed by specific stress-induced subsets of activated cardiac fibroblasts. Furthermore, it remains to be understood whether eliminating an entire cellular compartment is the ideal strategy for treating a disease such as heart failure. While complete elimination of cancer cells is the established goal in oncologic settings, this may not be the case for other clinical conditions. Activated cardiac fibroblasts cannot be defined as a solely pathological and maladaptive cell state, as they also have critical roles in tissue repair following injury or stress. Closely related, the timing for fibroblast-ablating immunotherapies is a crucial factor that remains to be determined as well. In the case of MI, for example, a fine balance must be struck between early intervention that may potentially interfere with the reparative phase that prevents myocardial rupture and late intervention when myocardial fibrosis may be too extensive. Recent single-cell studies have carefully characterized the fibroblast temporal response following cardiac injury [24, 25]. Studies of this nature will aid in shedding light on the complexity of the temporal fibroblast transcriptional response and may better guide the development of fibroblast-targeting therapies.

### Fibroblast cellular crosstalk

The homeostasis of the heart, or that of any organ, relies on a complex crosstalk between different cell types with specialized functions. Recent advances in single-cell ‘omics’ approaches have highlighted the heterogeneity of the cellular composition of the heart, revealing multiple subsets of cardiomyocytes, fibroblasts, endothelial and immune cells with diverse developmental origins and molecular properties [20]. Sophisticated cell-to-cell interactions between stromal, muscle, immune and vascular cells are known to be critical for tissue maintenance and repair. Cardiac fibroblasts are an extremely secretory cell type, and beside producing ECM, they secrete a multitude of factors, including cytokines and chemokines. These factors can contribute to fibroblast activation, as well as modulating other cellular compartments via crosstalk. For example, a previous study has highlighted the role of the cytokine Interleukin 11 (IL11) in activating a profibrotic response *in vivo* [42]. Factors secreted from activated fibroblasts can act as feedforward regulators to influence the activated fibroblast state itself, as well as by acting as amplifiers of the stress-response by signaling on receptors expressed in non-fibroblast cells. The combination of neutralizing antibodies to target specific stress-activated secreted factors, genetic models to specifically delete these factors in fibroblasts, and single-cell assays to capture the transcriptional response in other cell types will accelerate our mechanistic understanding of how the fibroblast secretome can influence disease pathogenesis.

### Is targeting cardiac fibroblasts sufficient to treat heart disease?

Our current understanding of how other cardiac cell compartments influence fibroblast cell states via cell-to-cell communication is limited. This is particularly important as we envision therapies that aim to substantially improve heart disease by targeting activated fibroblasts. Even if we would be able to safely and effectively decrease or eliminate activated fibroblasts, would this be sufficient to treat chronic heart disease? If another cellular compartment that provides a critical upstream signal for fibroblast activation remains unaffected, will the remaining quiescent fibroblasts keep transitioning to an activated state in the context of this chronic stress? Here again, single cell approaches will be pivotal in helping to identify specific cell compartments that can engage in direct communication with fibroblasts to affect heart disease outcomes. A better understanding of the molecular mechanisms driving cellular crosstalk will aid in designing therapies that most effectively target fibroblast activation in heart disease.

### Exploiting the momentum in cancer research

It is becoming increasingly clear that cancer-associated fibroblasts (CAFs) are a key component of the tumor microenvironment with many diverse functions, including remodeling of the ECM that can in turn modulate tumor stiffness and facilitate tumor progression, mediating reciprocal signaling interactions with cancer cells, and facilitating crosstalk with immune cells [43, 44]. As such, CAFs represent a potential target for optimizing therapeutic strategies against cancer. As the gene-programs modulating CAF activity within the tumor microenvironment might in part overlap with those governing fibroblast stress-responses in cardiac tissue, we believe that future findings regarding the biology of CAFs may be leveraged to for the development of new and more broadly applicable anti-fibrotic therapies.

## Perspectives

- Due to the lack of therapies that directly target excessive fibroblast activation, the global health burden of fibrotic disorders including cardiac fibrosis is strikingly high [45].
- Ongoing efforts to uncover the complex transcriptional and epigenomic regulation of cardiac fibroblasts activation with technologies such as single-cell genomics are increasing our understanding of the mechanistic basis of fibroblast activation.
- Such studies may ultimately inform novel therapeutic targets and strategies to treat various forms of heart disease that feature maladaptive remodeling of the fibroblast states and tissue fibrosis.

## Open Access

Open access for this article was enabled through a transformative open access agreement between Portland Press and the University of California.

## Abbreviations

ASO: antisense oligonucleotide
CAFs: cancer-associated fibroblasts
CAR: Chimeric antigen receptor
CHD: congenital heart disease
DCM: dilated cardiomyopathy
FAP: fibroblast activation protein
HCM: hypertrophic cardiomyopathy
HFpEF: heart failure with preserved ejection function
LNP: lipid nanoparticle
MI: myocardial infarction
PROseq: precision nuclear run-on sequencing
SMA: smooth muscle actin
TAC: transverse aortic constriction
TFs: transcription factors
TGF-β: transforming growth factor-β
